# Blood phenylalanine reduction reverses gene expression changes observed in a mouse model of phenylketonuria

**DOI:** 10.1038/s41598-021-02267-2

**Published:** 2021-11-24

**Authors:** Rachna Manek, Yao V. Zhang, Patricia Berthelette, Mahmud Hossain, Cathleen S. Cornell, Joseph Gans, Gulbenk Anarat-Cappillino, Sarah Geller, Robert Jackson, Dan Yu, Kuldeep Singh, Sue Ryan, Dinesh S. Bangari, Ethan Y. Xu, Sirkka R. M. Kyostio-Moore

**Affiliations:** 1 Genomic Medicine Unit, Sanofi, Framingham, MA USA; 2 Translational Sciences, Sanofi, Framingham, MA USA; 3 Pre-Development Sciences NA, Analytical R&D, Sanofi, Framingham, MA USA; 4 Global Discovery Pathology, Sanofi, Framingham, MA USA; 5 Excision BioTherapeutics, Cambridge, MA USA

**Keywords:** Gene therapy, Metabolic disorders, Proteomics, Transcriptomics

## Abstract

Phenylketonuria (PKU) is a genetic deficiency of phenylalanine hydroxylase (PAH) in liver resulting in blood phenylalanine (Phe) elevation and neurotoxicity. A pegylated phenylalanine ammonia lyase (PEG-PAL) metabolizing Phe into cinnamic acid was recently approved as treatment for PKU patients. A potentially one-time rAAV-based delivery of PAH gene into liver to convert Phe into tyrosine (Tyr), a normal way of Phe metabolism, has now also entered the clinic. To understand differences between these two Phe lowering strategies, we evaluated PAH and PAL expression in livers of PAH^enu2^ mice on brain and liver functions. Both lowered brain Phe and increased neurotransmitter levels and corrected animal behavior. However, PAL delivery required dose optimization, did not elevate brain Tyr levels and resulted in an immune response. The effect of hyperphenylalanemia on liver functions in PKU mice was assessed by transcriptome and proteomic analyses. We observed an elevation in Cyp4a10/14 proteins involved in lipid metabolism and upregulation of genes involved in cholesterol biosynthesis. Majority of the gene expression changes were corrected by PAH and PAL delivery though the role of these changes in PKU pathology is currently unclear. Taken together, here we show that blood Phe lowering strategy using PAH or PAL corrects both brain pathology as well as previously unknown lipid metabolism associated pathway changes in liver.

## Introduction

Phenylketonuria (PKU) is a genetic deficiency of phenylalanine hydroxylase (PAH), a liver enzyme that catalyzes hydroxylation of phenylalanine (Phe) to tyrosine (Tyr)^1,2^. In the absence of PAH, elevated levels of phenylalanine (Phe) accumulate in blood and subsequently in brain causing severe neurotoxicity^3,4^. Currently, patients are managed with Phe restricted diet starting soon after birth and have been instrumental in preventing intellectual disability^5,6^. However, Phe-restriction is a life-long requirement and due to severe dietary restrictions, the compliance among teen and adult patients has been poor resulting in various neurological and neuropsychological symptoms^4,7–9^. Some patients with milder forms of PKU can be treated with a synthetic form of a cofactor tetrahydrobiopterin (BH4) (Sapropterin dihydrochloride) that acts as a pharmacological chaperone to stabilize PAH^10,11^. This has been efficacious in lowering blood Phe levels and has also demonstrated improvement in neurological outcomes such as reduction in ADHD symptoms^10–12^. Another therapy recently approved consists of an enzyme substitution therapy using a PEGylated form of non-mammalian phenylalanine ammonia lyase (PAL) that metabolizes Phe into trans-cinnamic acid^13–15^. This therapy provides significant reduction in blood Phe levels but appears to be less efficacious on neurological endpoints^13^. Recently, a clinical trial evaluating rAAV based PAH gene delivery to liver was initiated as a first attempt to provide more sustained restoration of natural Phe metabolizing pathway^16^. In parallel, multiple preclinical studies are ongoing using various strategies, including mRNA delivery, and rely on either PAL or PAH production to reduce blood Phe levels^16–19^. In light of these two Phe lowering strategies, the goal of our study was to compare PAH and PAL expression on brain and liver functions after gene delivery via rAAV vectors into a PKU mouse model. Our data demonstrated that both PAH and PAL corrected various brain efficacy endpoints and the nest building behavior confirming that normalizing Phe levels can reverse most of the pathologies in this model. Our data also highlights previously unknown irregularities in overall lipid metabolism in the livers of PAH^enu2^ mice that were corrected by normalization of Phe levels with PAH or PAL.

## Results

### Both PAH and PAL gene transfer to liver reduces blood phenylalanine levels but exhibits subtle differences

**Figure 1:**
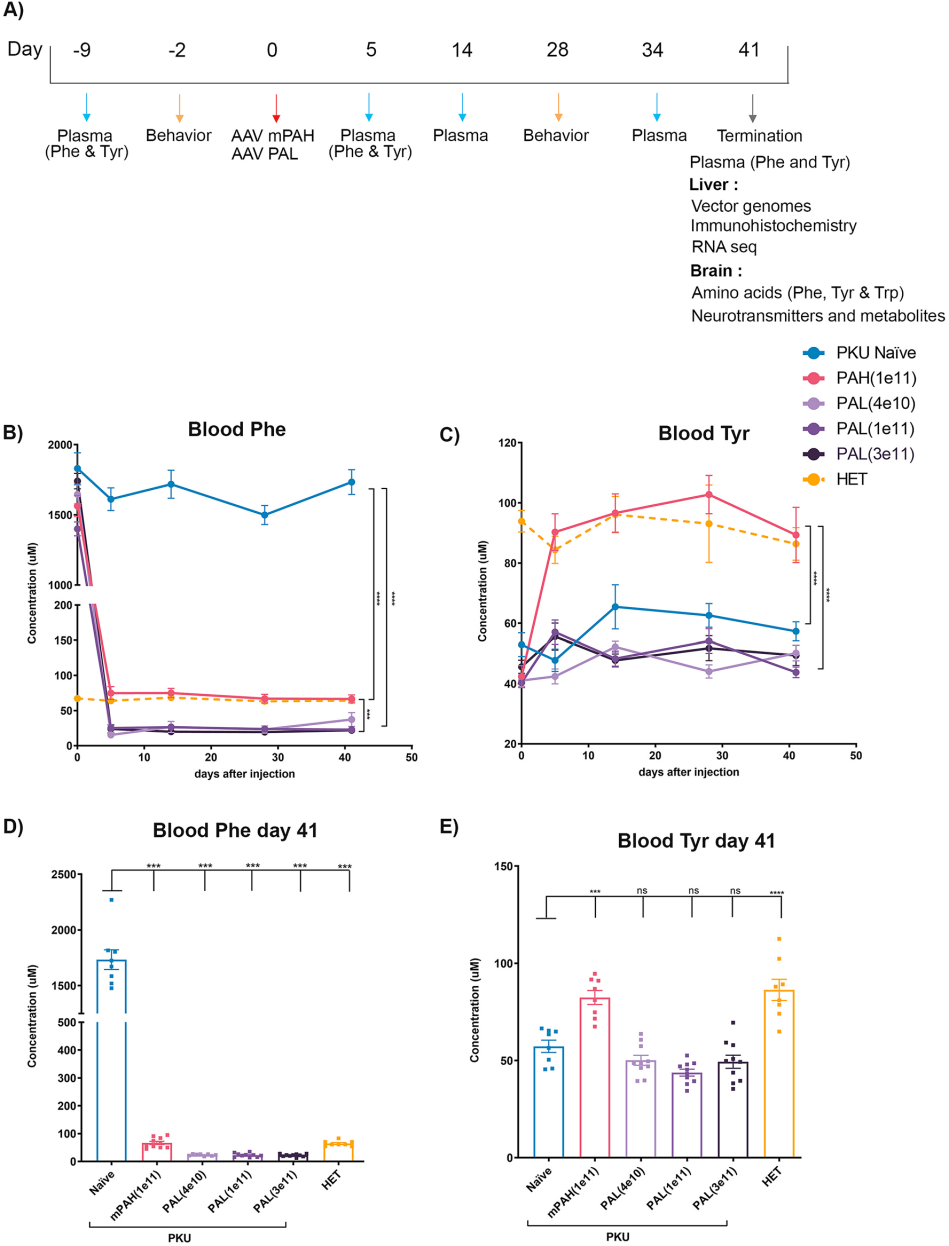
Effect of PAH and PAL gene transfer on blood Phe and Tyr levels after gene delivery to PAH^enu2^ mice. **(A)** Schematic of in-vivo study design. PAH and PAH encoding AAV vectors were administered by one-time IV delivery on Day 0. **(B)** Blood Phe levels were measured in naïve PAH^enu2^ mice, PAH^enu2^ mice administered with PAH (1e11 vg) or PAL (4e10, 1e11 or 3e11 vg/mouse) and HET mice at day 9 (baseline) and days 5, 14, 34 and 41 post treatment. Significant reduction in blood Phe levels was observed in both PAH and PAL in PAH^enu2^ mice as compared to naïve PAH^enu2^ mice. PAH^enu2^ PAL treated mice show lower Phe than HET mice while PAH treated mice maintain Phe levels comparable to HET mice. **(C)** Significant increase in blood Tyr to levels comparable to HET mice were achieved in PAH treated mice but not in PAL treated mice. Two-way ANOVA Mixed effect analysis was performed, ***p < 0.001. **(D)** Blood Phe continued to remain low in PAH and PAL PAH^enu2^ mice until the end of the study. **(E)** Blood Tyr remained normal in PAH PAH^enu2^ mice till the end of the study. Treatment groups consisted of n = 8–10 animals. Statistics by One-way ANOVA Tukey’s multiple comparison, ***p < 0.001.

To provide sustained expression of murine PAH (PAH) and PAL in the livers of PAH^enu2^ mice, rAAV vectors encoding either murine PAH (PAH) or PAL were administered by one-time IV delivery. The PAH vector was injected at a dose of 1e11 vg/mouse based on prior efficacy studies while PAL vector was administered at various doses (4e10 vg, 1e11 vg or 3e11 vg per mouse) to establish an optimal dose. Blood Phe and Tyr were measured at regular intervals through the study (days 9, 5, 14, 34 and 41). Nesting behavior was assessed pre (day 2) and post treatment (day 28). Livers and brains were harvested on day 41 for various analysis (Fig. 1A). Treated animals were compared to heterozygous PAH^enu2^ mice (HET) for all the analysis as these mice are phenotypically normal and have blood Phe levels comparable to WT mice.

The blood Phe levels were reduced to normal range (75 ± 31 µM) in PAH treated PAH^enu2^ mice and were comparable to non-phenotypic HET mice (64 ± 13 µM) within 1 week of vector administration and continued to remain so throughout the study (Fig. 1B,D). The blood Phe levels in PAL treated PAH^enu2^ mice were reduced to levels lower than those observed in normal HET mice (64 ± 13 µM) at all the given doses (19 ± 7 µM at 4e10 vg, 28 ± 10 µM at 1e11 vg and 24 ± 17 µM at 3e11 vg) as seen in Fig. 1B. To understand if lowering the dose of PAL would avoid the over-correction of Phe levels, a second independent study was performed (Supplemental Fig. S1). Since the lowest efficacious dose in the previous study was 4e10 vg, a dose of 1e10 vg was included in this study. However, blood Phe levels (894 ± 673 µM) did not achieve normal levels for 7 out 8 PAL PAH^enu2^ mice treated at 1e10 vg dose (Supplementary Fig. S1B). Blood Tyr levels increased to normal levels from 42 ± 12.1 to 92.6 ± 18.9 µM with PAH expression within 1 week of treatment and continued to stay in the normal range throughout the study (Fig. 1C,E). As expected, similar increase in Tyr levels was not observed after PAL treatment since this enzyme converts Phe to trans-cinnamic acid (Fig. 1C,E).

Vector genome analysis showed a dose-dependent increase of PAL vector in the livers; at 1e11 vg/mouse dose, comparable levels of vector genomes were observed for both PAH and PAL vectors in the livers (Supplemental Fig. S2A). Measurement of liver enzymes Alanine transaminase (ALT) and Aspartate transaminase (AST) in the serum did not show any significant treatment mediated adverse effects on the liver (Supplemental Fig. S2B,C). However, histopathological evaluation of liver tissue by H&E staining showed that all animals injected with PAL vector had a variably dense perivascular and portal aggregates of mononuclear inflammatory cells and the median scores for inflammatory cells exhibited dose related trends (Supplemental Fig. S2D). Among these mononuclear inflammatory cells, plasma cells were the predominant population. The nature of these cells as B-cell infiltrates was further supported by increased expression of immunoglobin genes in the liver RNA-seq analysis (Supplemental Fig. S2E). These findings are most likely attributable to bacterial origin of PAL as PAH treatment with similar vector design did not show any histopathological abnormalities.

### Delivery of PAH and PAL genes to livers normalizes amino acid imbalance, brain neurotransmitter levels and corrects behavioral defect in PAH^enu2^ mice

**Figure 2:**
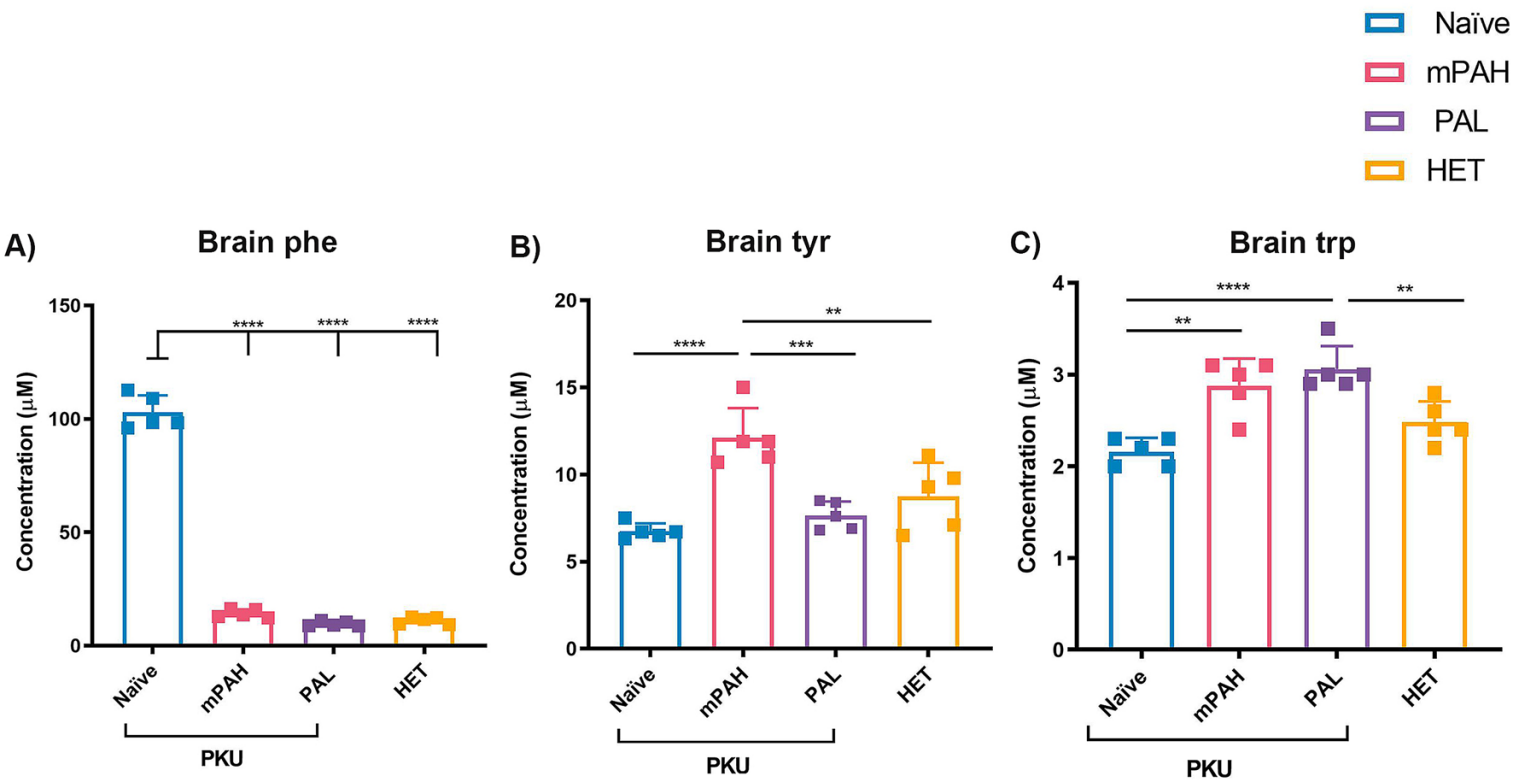
Brain amino acid levels post PAH or PAL administration. **(A)** Brain Phe is reduced to normal levels in PAH or PAL treated PAH^enu2^ mice. **(B)** Brain Tyr levels are restored to normal in PAH PAH^enu2^ mice but not in PAL PAH^enu2^ mice. **(C)** Brain Trp is increased in both PAH and PAL treated PAH^enu2^ mice. N = 5 per group were used for analysis. Control mice and mice treated with 1e11 vg/mouse of PAH or PAL vector were terminated on day 41 and perfused with PBS. *Naïve* untreated PAH^enu2^ mice, *PAH or PAL*, treated PAH^enu2^ mice, *HET* untreated HET mice. One-way ANOVA Tukey’s multiple comparison, **p < 0.01 and ****p < 0.0001.

**Figure 3:**
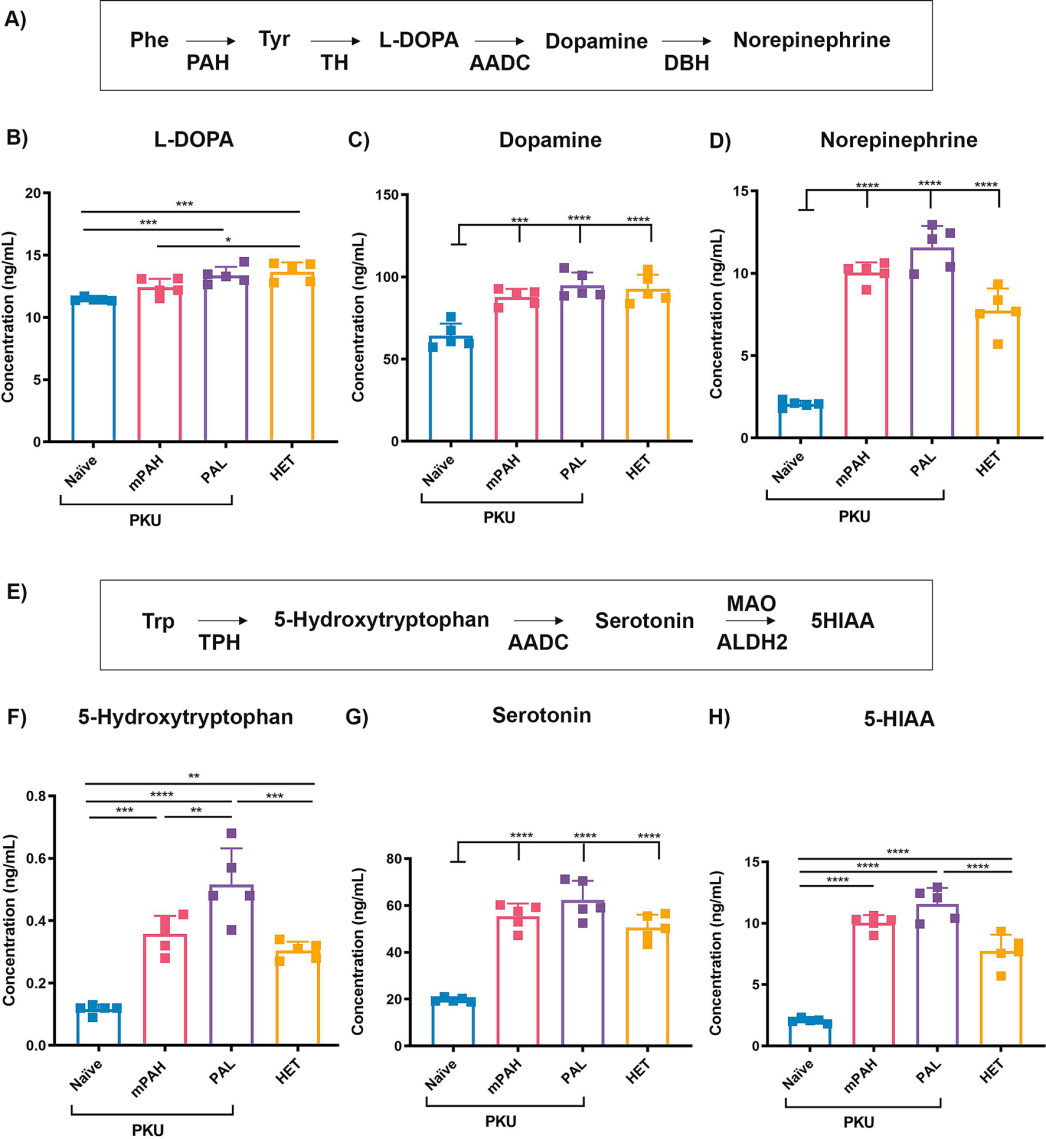
Restoration of brain neurotransmitter levels after PAH or PAL administration. **(A)** Schematic of major steps involved in synthesis of neurotransmitters dopamine and norepinephrine. (**B)** Dopamine precursor L-DOPA was significantly higher in PAL PAH^enu2^ and HET brains as compared to the naïve PAH^enu2^ brains. Significant increases in **(C)** dopamine and **(D)** norepinephrine were observed in both the PAH and PAL PAH^enu2^ mouse brains as compared to the naïve mice. **(E)** Schematic of serotonin synthesis and downstream metabolite 5-hydroxyindolacetic acid (5-HIAA). Serotonin precursor 5-hydroxytryptophan **(F)**, serotonin **(G)** and 5-HIAA **(H)** were all restored to levels similar to HET mice after PAH and PAL administration. N = 5 per group were used for analysis. Control mice and mice treated with 1e11 vg/mouse of PAH or PAL vector were terminated on day 41 and perfused with PBS. Group abbreviations: PKU naïve, untreated PAH^enu2^ mice; PKU PAH or PAL, treated PAH^enu2^ mice; HET, untreated HET mice. One-way ANOVA Tukey’s multiple comparison, *p < 0.05, **p < 0.01, ***p < 0.001 and ****p < 0.0001.

The PAH and PAL treatment groups dosed at 3e11 were used for biochemical brain analyses (n = 5 per group). High brain Phe in PKU naïve mice (102.9 ± 7.5 µM) was reduced to normal levels after PAH (14.18 ± 1.82 µM) and PAL treatment (9.62 ± 1.07 µM) while the increase in brain Tyr from 6.74 ± 0.46 µM to 12.1 ± 1.71 µM was observed only in PAH treated mice (Fig. 2). No significant differences were observed in brain Tyr in the PAH^enu2^ naïve and HET mice despite 30–40% higher blood Tyr levels in HET mice. Brain tryptophan (Trp) levels also increased from 2.16 ± 0.15 to 2.88 ± 0.29 µM with PAH treatment and to 3.06 ± 0.25 µM with PAL treatment (Fig. 2). Analysis of brain neurotransmitter levels showed a marked increase in L-DOPA, dopamine and norepinephrine levels in both PAH and PAL treated mice (Fig. 3B–D). Similarly, a significant increase of 5-hydroxytryptophan, serotonin and serotonin metabolite 5-hydroxyindolactetic acid (5-HIAA) levels were observed after expression of either PAH or PAL in comparison to naïve PKU mice (Fig. 3F–H).

**Figure 4:**
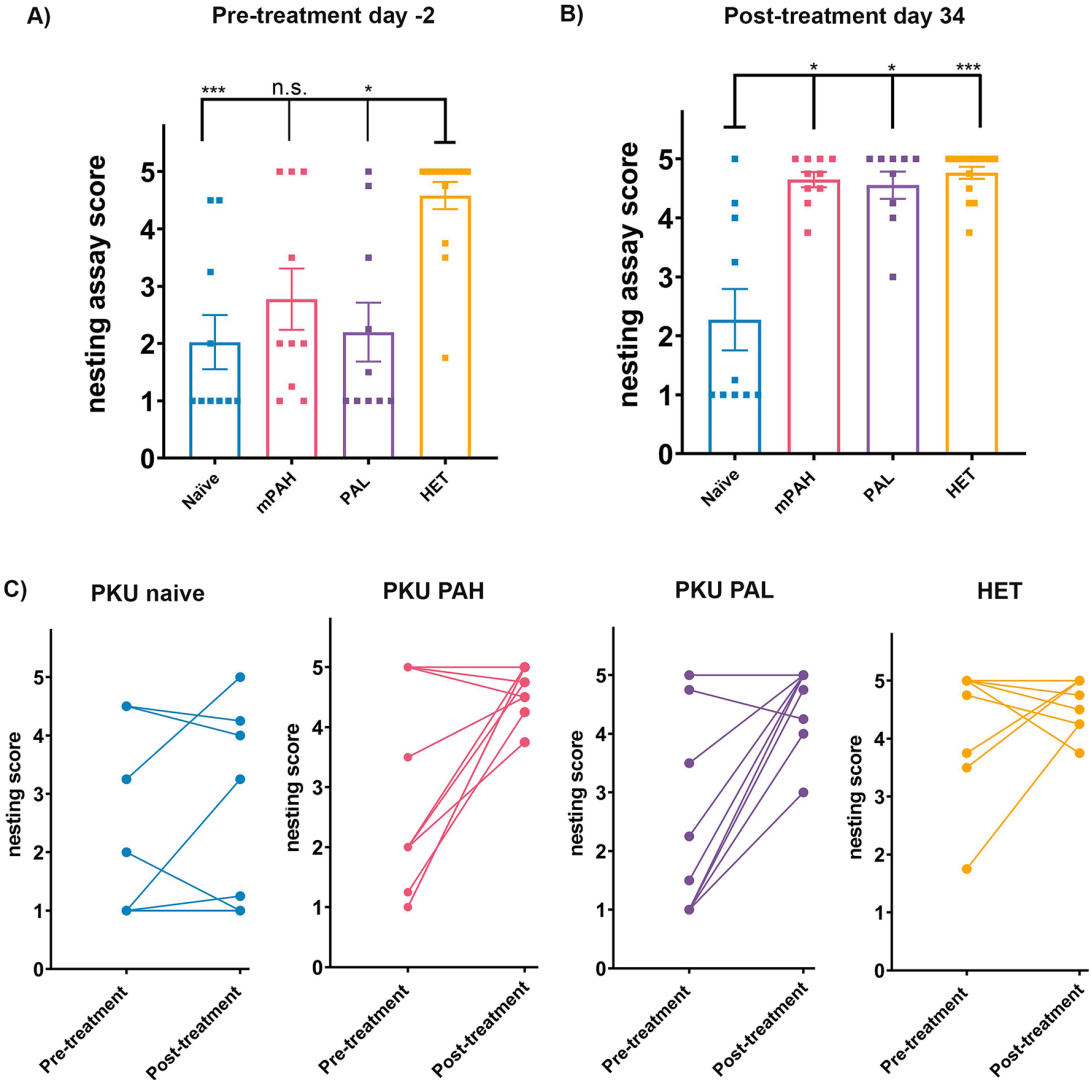
Improvement in nesting behavior in PAH and PAL PAH^enu2^ mice. **(A)** Baseline behavior measured 2 days prior to treatment and **(B)** improved at day 34 post PAH and PAL treatment. **(C)** Tracking the nesting score of individual mice shows that most PAH and PAL treated mice perform better and receive a higher score in the post treatment nesting assay. Treatment groups consisted of n = 8–10 animals. Group abbreviations: PKU naïve, untreated PAH^enu2^ mice; PKU PAH or PAL, treated PAH^enu2^ mice; HET, untreated HET mice. Statistics by One-way ANOVA Kruskal–Wallis test followed by Dunn’s multiple comparison, *p < 0.05 and ***p < 0.001.

Change in neurotransmitter levels has been associated with neurobehavioral phenotypes in PKU patients and mice. We measured the ability of PAH^enu2^ and HET mice to build nests, a social behavior commonly seen in normal mice. Mice were scored on a scale of 1–5 based on their ability to form a nest. Baseline pretreatment nesting behavior was significantly lower in most naive PAH^enu2^ mice compared to the normal HET mice (Fig. 4A). After 34 days of treatment, a significant improvement was observed in nest building ability of PAH and PAL treated mice while untreated mice still performed poorly (Fig. 4B). The pre- and post-treatment scores for each individual mouse are shown in Fig. 4C.

### Transcriptome analysis of livers of PAH^enu2^ mice

**Figure 5:**
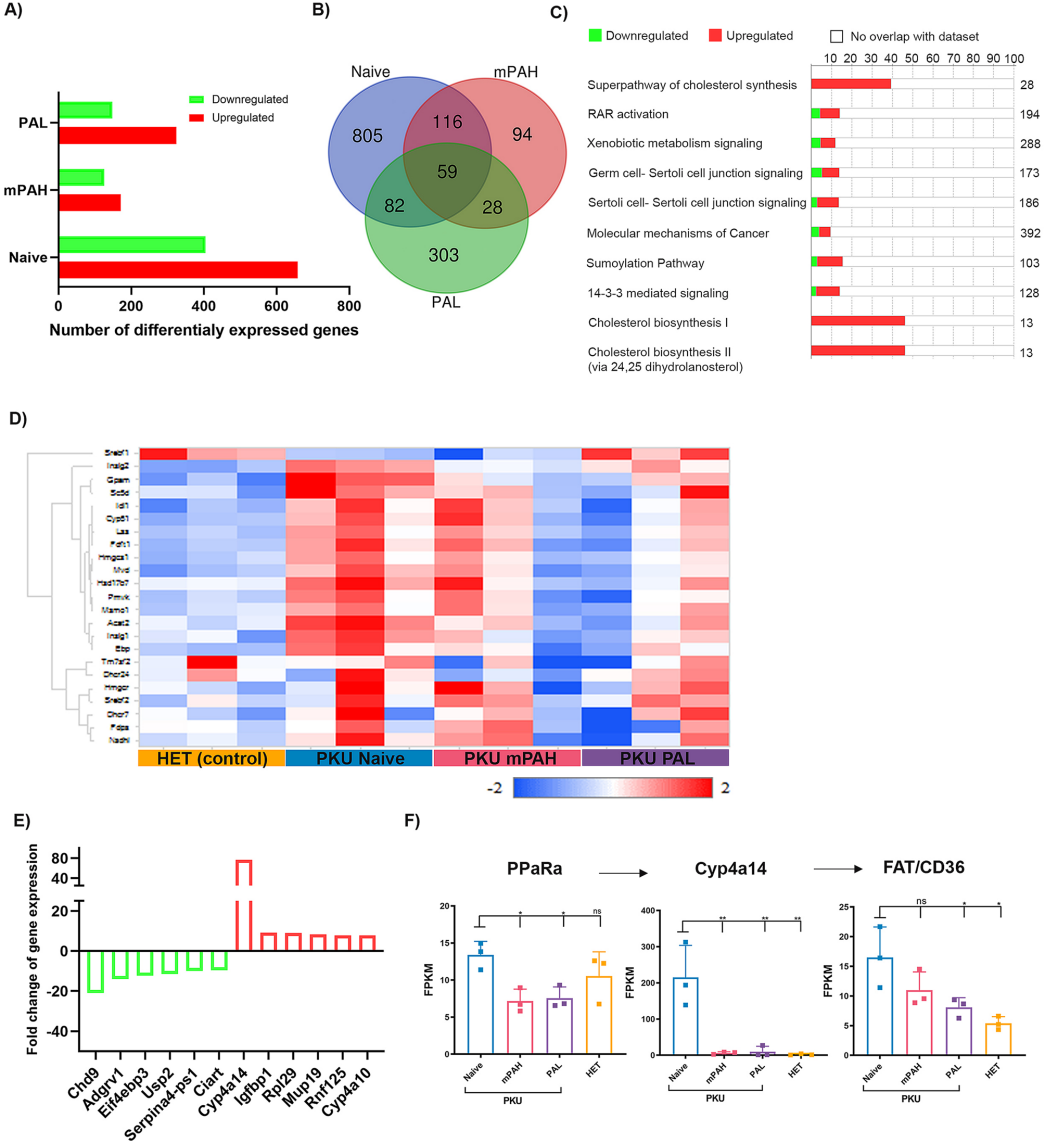
Gene expression changes in livers of PAH^enu2^ mice. **(A)** Number of differentially expressed genes in naïve, PAH and PAL treated PAH^enu2^ mice as compared to HET mice (HET). **(B)** Venn diagram showing number of DEG common and unique to each group. The majority of DEG in PAH^enu2^ mice are corrected after PAH or PAL treatment. **(C**) Top 10 pathways affected in the liver of PAH^enu2^ mice as compared to HET mice analyzed by Ingenuity pathway analysis. Superpathway of cholesterol biosynthesis was the most affected pathway in the liver of PAH^enu2^ mice. **(D)** Heatmap showing the expression of genes involved in multistep cholesterol biosynthesis pathway. Most genes involved in cholesterol biosynthesis are upregulated in untreated PAH^enu2^ mice (PKU naive). The expression profile of PAH and PAL treated mice shares similarity to naïve HET mice (HET, control). Heatmap was generated using QIAGEN Omicsoft studio version 10.2.4.7. **(E)** Top 6 upregulated and downregulated genes in PAH^enu2^ mice as compared to naïve HET mice. **(F)** Modest increase in expression of PPARα, activator of Cyp4a14 and FAT, a downstream effector of high Cyp4a14 in PAH^enu2^ mice. Upregulation of Cyp4a14, PPARα and FAT is corrected in both PAH and PAL treated mice. N = 3 per group were used for analysis. One-way ANOVA Tukey’s multiple comparison, *p < 0.05 and **p < 0.01.

To obtain a more in-depth look at the disease mediated changes and the differences in treatment strategies, we performed high-throughput transcriptome sequencing (RNA-seq) analyses in the livers of PAH^enu2^ mice. Comparison of gene expression in the liver of naïve PAH^enu2^ mice to HET mice revealed 1062 differentially expressed genes (p < 0.05 and fold change ≥ 1.5) of which 658 genes were upregulated while 404 genes were downregulated. Treatment with PAH reduced the differentially expressed genes (DEG) to 297 (p < 0.05 and fold change ≥ 1.5) with 171 upregulated and 126 downregulated while PAL treated mice showed 472 differentially expressed genes (p < 0.05 and fold change ≥ 1.5) of which 324 were upregulated and 148 downregulated (Fig. 5A). Of the 1062 DEG in naïve PAH^enu2^ mice, expression of 887 genes was corrected by PAH treatment and 921 by PAL treatment suggesting that both treatments effectively corrected majority of the gene expression changes (Fig. 5B). On comparing the gene expression changes between the two treatment groups, we observed a significant upregulation of genes immunoglobin encoding genes in PAL treated mice that was not observed in PAH treated mice (Supplementary Fig. S2E). Mononuclear inflammatory cells were considered the source of these upregulated genes.

Next, we performed Ingenuity Pathway Analysis (IPA) to study specific pathways impacted by the differentially expressed genes (DEG). Pathways associated with cholesterol biosynthesis were found to be the most affected in untreated PAH^enu2^ mice (Fig. 5C). Modest but consistent upregulation was observed for the majority of genes at various steps of cholesterol biosynthesis pathway in naïve PAH^enu2^ mice (Fig. 5D). This included small upregulation of sterol regulatory element binding transcription factor 2 (SREBF2), a master-regulator of the cholesterol pathway while sterol regulatory element binding transcription factor 1 (SREBF1), more involved in regulation of fatty acid synthesis was slightly downregulated (Fig. 5D, Supplementary Fig. S3A). Treatment with PAH and PAL lead to the correction of most genes involved in cholesterol biosynthesis to normal levels suggesting that the changes in cholesterol biosynthesis pathway were linked to the disease (Fig. 5D, Supplementary Fig. S3A). RT-PCR validation of key genes of cholesterol biosynthesis corroborated the findings from RNA-seq (Supplementary Fig. S3A).

The top 6 upregulated and downregulated genes in PAH^enu2^ mice as compared to normal HET mice are shown in Fig. 5E. One striking observation was a massive upregulation (76-fold) of Cyp4a14 in naive PAH^enu2^ mice, a gene encoding a cytochrome P450 monooxygenase (Fig. 5E). The overexpression of this gene and Cyp4a10, another P450 mono-oxygenase were confirmed by RT-PCR (Supplementary Fig. S3B). No upregulation of Cyp4a14 and Cyp4a10 were detected in the PAH and PAL treatment cohorts (Fig. 5F, Supplementary Fig. S3B). To observe potential upstream and downstream impact to Cyp4a14 upregulation, we analyzed the expression of Peroxisome proliferator-activated Receptor α (PPARα) and Fatty acid translocase (FAT)/CD36. Both genes were upregulated in naïve PAH^enu2^ mice and subsequently downregulated with PAH and PAL treatments (Fig. 5F, Supplementary Fig. S3B).

### Proteomic analysis of livers of PAH^enu2^ mice

**Figure 6:**
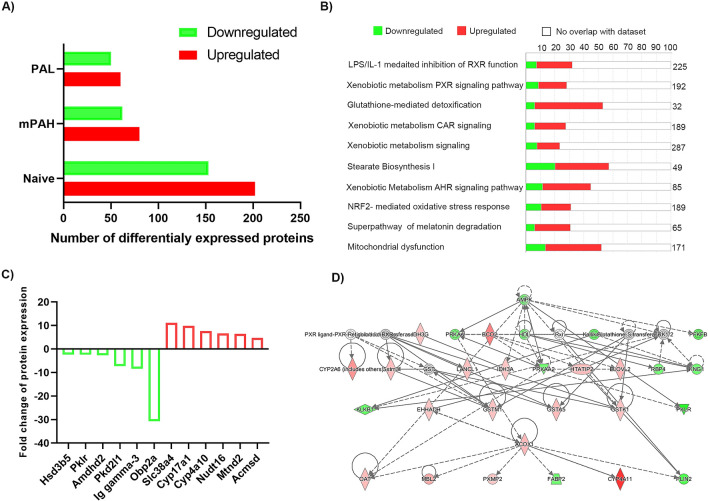
Changes in protein levels in livers of PAH^enu2^ mice. **(A)** Number of differentially expressed proteins in naïve, PAH and PAL treated PAH^enu2^ mice as compared to HET mice. **(B)** Top 10 pathways affected in the livers of PAH^enu2^ mice as compared to HET mice analyzed by Ingenuity pathway analysis. Pathways linked to lipid metabolism are the most affected pathways in the liver of PAH^enu2^ mice. **(C)** Top 6 upregulated and downregulated proteins in PAH^enu2^ mice as compared to HET mice. **(D)** Network analysis identified Lipid metabolism as the top network in the dataset with Cyp4a11 (Cyp4a10 in mouse) at the bottom of the hierarchy. Network analysis figure was generated using Ingenuity Pathway analysis (IPA) (QIAGEN Inc., https://www.qiagenbioinformatics.com/products/ingenuitypathway-analysis).

To confirm that the changes in the liver gene expression profiles translated to changes at the protein level, we performed another study to analyze the proteome of the livers of PAH^enu2^ mice. Proteomic analysis was performed on PAH^enu2^ mice, PAH^enu2^ mice treated with either PAH or PAL at the dose of 1e11 and HET mice (Supplementary Fig. S1A). A total of 355 proteins (202 upregulated and 202 downregulated) were differentially expressed in the livers of PAH^enu2^ mice as compared to HET mice while PAH and PAL treated PKU mice showed fewer deregulated proteins (Fig. 6A). Similar to the RNA-seq results, Cyp4a10 protein was among the top upregulated proteins in PAH^enu2^ mice along with Cyp17a1 which is mainly involved in steroid biosynthesis (Fig. 6C). Cyp4a14 was also ninefold upregulated (p value = 0.051) in PAH^enu2^ mice but was excluded from our analysis as it was slightly above our cutoff criteria of statistical significance (p ≤ 0.05). Other top upregulated proteins included Mtnd2, a mitochondrial protein coding for NADH dehydrogenase and Acmsd, an enzyme involved in NAD^+^ regulation and maintaining mitochondrial homeostasis. The IPA analysis of deregulated proteins in PAH^enu2^ mice relative to HET mice identified LPS/IL-1 mediated inhibition of RXR function, (downstream function of this pathway is lipid and xenobiotic metabolism), xenobiotic metabolism PXR signaling and glutathione mediated detoxification among the top affected pathways (Fig. 6B). The expression of top three proteins in each of these affected pathways is shown in Supplementary Fig. S4A–C). All these proteins were upregulated in PAH^enu2^ mice and either normalized or trending towards normalization post PAH and PAL treatment. We also observed modest changes in various proteins involved in cholesterol biosynthesis (Supplementary Fig. S4D) consistent with the gene expression results above. Next, we performed a network analysis to understand the interaction between the differentially expressed proteins in our data set. This analysis assumes that highly connected networks are biologically significant. The network with highest score was lipid metabolism suggesting that the greatest number of differentially expressed proteins in the liver of PAH^enu2^ mice were functionally involved in lipid metabolism. The hierarchical clustering of this network with Cyp4a10 at the bottom of hierarchy is shown in Fig. 6D. Proteins downregulated in the dataset are shown in green and upregulated proteins are in red.

## Discussion

Reduction of blood Phe levels has been the main treatment goal in PKU patients to minimize toxic Phe effect on brain^16,19,20^. The current standard of care to achieve this is the consumption of low Phe diet which is initiated soon after birth to prevent severe brain damage. However, compliance with strict diet steadily decreases with age and among teens and adults, the majority of the patients have higher than recommended Phe levels^8,9^. The only other approved therapy for severe PKU patients currently is PEGylated PAL that is a non-mammalian enzyme converting Phe into trans-cinnamic acid^13–15^. Though efficacious, the therapy requires daily s.c. administrations, long titration period and the patients often develop immune responses both the PEG and PAL^21^. Multiple other therapies are currently in development including rAAV-based PAH gene replacement to provide a normal Phe metabolizing pathway in a more sustained and stable manner^16^.

To better understand potential differences between the two strategies for Phe metabolism and to study the effects of elevated Phe on liver and brain, we delivered PAL and PAH genes into livers of PAH^enu2^ mice, a model of human PKU. Our data demonstrated that viral mediated gene transfer of either gene resulted in reduced blood Phe levels, though the use of PAL over-corrected when high doses were administered. Hence the use of PAL will require careful optimization to obtain normal blood Phe levels as is currently performed for PEGylated PAL in the clinic^15^. This is not surprising as PAL is not subject to allosteric regulation by blood Phe levels. In contrast, multiple studies have demonstrated fine-tuning of PAH activity by Phe binding to N-terminal regulatory domain of PAH^22,23^. At lower Phe levels, the enzyme is maintained as a less active dimer form while with increasing Phe levels the enzyme is driven into a more active tetramer form^22,24^. As expected, only PAH treatment increased the Tyr levels in the blood.

The mechanism by which high Phe causes neurotoxicity in PKU is unclear. Potential explanations include reduced amino acid transport into brain and subsequent reduction in brain neurotransmitter levels^4,25^. High blood Phe competes with the transport of large neutral amino acids (including Tyr and Trp) into brain causing overall lowered levels of these amino acids in brain^4,25^. We observed this more for Trp than for Tyr and this may have been due to the use of normal rodent diet. High Phe levels have also been reported to inhibit protein production and enzyme activities, particularly the enzymes involved in neurotransmitter synthesis^25^. Our brain analyses of animals treated with PAL and PAH vectors showed that both treatments normalized brain Phe levels due to reduction of Phe in the blood. Both treatments also corrected brain Trp levels indicating a better large amino acid transport to brain. The LAT1 transporter has been reported to have high affinity to Phe resulting in lowered amino acid transport of large neutral amino acids in the presence of high Phe^26^. Only PAH provided elevated Tyr in the brain as expected due to higher Tyr measured in blood. Despite animals being fed the normal diet, the PAL treated mice exhibited low blood Tyr levels which corresponded to lower-than-normal Tyr in the brain (Figs. 1D,E, 2B). This was expected to result in lower dopamine levels since Tyr is a substrate for dopamine synthesis. However, both PAL and PAH treatments normalized brain dopamine levels suggesting that Tyr deficiency is not the main cause for reduced brain dopamine levels in PKU mice. Other products in the dopamine pathway such as L-DOPA and norepinephrine were also consistently increased by both treatments (Fig. 3B,D). While the low Tyr levels in the PAL treatment group did not seem to be limiting for dopamine synthesis the higher Tyr in PAH treatment group may be beneficial during suboptimal Phe reduction. Interestingly, serotonin and its intermediates and breakdown products had a much higher magnitude of correction with PAH and PAL than those in the dopamine pathway. Furthermore, there was a trend of PAL treatment providing higher levels of neurotransmitters which may have been due to slightly lower blood and brain Phe levels (Fig. 3). This suggests that neurotransmitter synthetizing enzymes are highly sensitive to Phe levels in the brain.

Unlike the PAH delivery, the delivery of PAL vector resulted in detectable immune response in liver. An infiltration of inflammatory cells, mostly consisting of B-cells, was observed in all PAL treated animals using histopathology. The nature of these cells as B-cells was also supported by RNA seq analysis of liver that showed upregulation of immunoglobulin genes. The immune response was not unexpected since PAL is a bacterial protein. The material used in the clinic has been PEGylated to reduce the immune response. Despite this, high and sustained levels of antibodies both to PAL and PEG were observed in PKU patients and hence, PKU patients using PEG-PAL (Palynziq) are required to carry EpiPen as a precautionary measure^21,27^.

Though neurotoxicity is the hallmark of PKU pathology, we attempted to understand the effect of hyperphenylalanemia in the liver, the major site for PAH expression. Both the transcriptome and proteomics data revealed changes in large number of genes (Figs. 5A, 6A). Most of these changes were reversed post Phe normalization by PAH and PAL treatments suggesting that these changes were caused by elevated Phe levels and hence were disease specific changes (Figs. 5A,B, 6A). Both analyses demonstrated the cholesterol synthesis pathway was upregulated in PKU with elevated expression levels of many enzymes involved in this complex multistep pathway (Fig. 5C,D, Supplementary Fig. S4D). These included SREB2, the master-regulator of the pathway while the SREB1, controlling the fatty acid synthesis was reduced. This upregulation could be in response to the low total cholesterol levels in sera of PAH^enu2^ mice and PAH-KO mice observed by us and others^28,29^. In PKU patients, serum cholesterol, HDL, LDL are lower than in healthy controls^30^. Similarly, long-chain unsaturated fatty acids levels such as docosahexaenoic acid (DHA) and arachidonic acid (AA) have been reported to be lower in PKU patients^30^. The lowered cholesterol has been proposed to be caused by the inhibition of Phe or its metabolites on HMG-CoA reductase and mevalonate 5-pyrophosphate decarboxylase^28,31^.

The highest degree of over expression in the PKU naïve mice livers was from CYP4A family (Cyp4a10, Cyp4a14 and Cyp17a1) of heme-containing monooxygenases that are largely involved in oxidation of lipids (Figs. 5E, 6C). Of these, Cyp4a10 and 14 are involved in microsomal oxidation of medium to long chain fatty acids specifically with a hydroxylated terminal ώ-carbon^32^. Upregulation of these Cyp proteins has not been previously reported in the liver though Cyp4a14 was elevated in the brains of PAHenu2 mice^33^. In the liver, Cyp4a10 and Cyp4a14 expression is known to be induced by a nuclear receptor peroxisome proliferator-activated receptor (PPARα), a ligand activated transcription factor that regulates lipid and lipoprotein metabolism. PPARα is mainly activated by elevated free fatty acids and upon activation it enhances hepatic lipid metabolism by upregulation of fatty acid translocase (FAT) /CD36 that functions to mediate the uptake of long chain fatty acids into the cell^32^. Fatty acids can subsequently be removed via increased peroxisomal and mitochondrial fatty acid beta-oxidation. Our data showed that the expression of both PPARα and FAT/CD36 were slightly increased in PAH^enu2^ mice suggestive of potential biological impact of Cyp4a10/Cyp4a14 upregulation (Fig. 5F). Substrates of Cyp4a10 (Cyp4a11 in humans), long chain poly unsaturated fatty acid (LC-PUFA) such as eicosapentaenoic acid (EPA) and docosahexaenoic acid (DHA) have been reported to be low in PKU patients suggesting possible downstream consequences of increased Cyp4a10 expression^34^. In-fact lower levels of LC-PUFA are also observed in children on unrestricted diet suggesting that these changes are specific to the disease and not merely a consequence of PKU diet^35^. Besides potential microsomal omega oxidation via increased Cyp4a14, enzymes involved beta oxidation, Acox1 and Acot4, were also increased by proteomics. Elevation in Cyp4a14 has also been reported in models of nonalcoholic fatty liver disease and shown to contribute to hepatic steatosis and nonalcoholic steatohepatitis by increased oxidative stress due to lipid accumulation^32^. While we observed an increase in expression of various glutathione transferases (Fig. 6B, Supplementary Fig. S4C), enzymes that protect cell from reactive species, it should be noted that liver pathology indicative of massive oxidative stress or consistent elevation of liver enzymes was not observed in untreated PAH^enu2^ mice. Furthermore, neither liver pathology nor oxidative stress and elevated liver enzymes have been reported in PKU patients. However, PKU mice exhibit poor growth which can be corrected with Phe reduction suggesting Phe effect on energy metabolism and lipid dysfunction^36^. Taken together, our data highlights a novel observation of family of Cyp4a protein upregulation that may increase uptake of fatty acids and their oxidation. Whether this may contribute to lowered sera cholesterol, HDL and LDL observed in PKU patients is currently unclear.

It should be noted that the changes in cholesterol and lipid levels in our PAH^enu2^ mouse study were obtained with animals fed with normal rodent diet during their entire life span. However, understanding the causative nature of changes in lipid and cholesterol levels in PKU patients is clearly more complex as the disease pathology, compliance with Phe-restricted diet, fluctuations in Phe levels and other underlying genetic factors all likely impact lipid levels^30,37–39^. It has been generally thought that the consumption of Phe-restricted diet consisting little animal derived products contributes to lower levels of lipids and cholesterol. However, even non-compliant PKU patients tend to have lower HDL levels^40^ suggesting that lipid alterations in PKU patients are influenced by disease pathology in addition to diet. Interestingly, compliant PKU patients also tend to have high rate of being overweight thought to be at partially caused by the use of protein substitutes and commercial low-Phe products with high carbohydrate content^37,38^. However, the effect of Phe on lipid metabolism with increase fatty acid uptake and metabolism may also play a role. Lastly, the impact of lipid alterations and increased bodyweights in PKU patients on various co-morbidities such as cardiovascular disease and atherosclerosis are important topics of discussion for the care of PKU patients and should be aided with better understanding the underlying disease.

In summary, our data demonstrated that Phe reduction could effectively be obtained with PAH or PAL expression and resulted in improved brain health in mice. Obtaining normal brain Phe levels appeared to be especially critical for restoring neurotransmitter synthesis while amino acid transport was less critical. Our data also highlighted novel changes in lipid metabolism pathways in the PKU liver indicating elevated Phe has profound effects in other organs beyond the known neurotoxicity.

## Materials and methods

### Gene delivery vector generation

The expression cassette was based on liver-specific promoter mTTR482, hybrid intron, polyadenylation sites (bovine growth hormone [BGH]) and has been described before^41^. These expression elements were modified to contain A1MB2 enhancer (2 copies alpha1-microglobulin) upstream of mTTR482 and an intron consisting of CBA (chicken beta actin)/rabbit beta globin hybrid intron). The vectors encoded either human codon-optimized murine PAH or Anabaena variabilis PAL. Both proteins contained 3xFLAG fusion (DYKDDDDK) for detection purposes (PAH, N-terminal fusion and PAL, C-terminal fusion). Additionally, the PAL vector contained 0.9 kb A1AT filler sequence downstream of BGH pA. Plasmid vectors were confirmed for PAH and PAL production in vitro (data not shown). Single-stranded recombinant AAV vectors with AAV2 ITRs and a liver tropic AAV capsid were generated using triple transfection method followed by CsCl2 purification (Univ. Massachusetts Gene Therapy Core). Vector lots were quantitated by qPCR to BGHpA^41^.

### Animal procedures

All animal procedures were approved by the Sanofi’s Institutional Animal Care and Use Committee (IACUC) in an animal facility accredited by Association for Assessment and Accreditation of Laboratory Animal Care International (AAALAC) and in compliance with ARRIVE guidelines (http://www.nc3rs.org.uk/page.asp?id=1357). A colony of PAH-deficient BTBR-PAH^enu2^ (PKU) mice was maintained at Taconic^42^. For both the studies (Fig. 1, Supplementary Fig. S1A), homozygous (n = 46–48) were divided into n = 8–10 per group and heterozygous male mice (n = 8–10) were obtained at 8–9 weeks of age and were housed in accordance with humane guidelines for animal care and use. Animals were individually caged and fed regular 16% protein diet. Vectors encoding PAH or PAL were administered by intravenous route via tail vein. For Phe measurement during the study, mice were anesthetized with isoflurane, blood was collected by retro-orbital sinus into EDTA collection tubes, spun and stored frozen until analysis. For termination, animals were euthanized humanely by CO_2_ by asphyxiation as per Sanofi IACUC protocols. Some animals were intracardially perfused by PBS before tissue collection. Liver and brain samples were collected and frozen at – 80 °C until or fixed in PFA for further analysis.

### Measurement of amino acids and neurotransmitters

Blood Phe and Tyr levels as well as brain amino acids (Phe, Tyr and Trp) measurement methods are described by Singh et al. For brain neurotransmitter quantitation, brains were processed as described in Ref.^43^ with minor modification described in Singh et al.^29^.

### Liver vector DNA quantitation and histopathology

#### Vector quantitation

Liver tissue was homogenized in 1 ml lysis buffer (100 mM Tris pH 8.5, 5 mM EDTA, 0.2% SDS, 200 mM NaCl) in 2 ml tubes with 1.4 mm ceramic beads using an Omni Ruptor—24 for 30 s at 5.65 m/s. Lysates were spun at max speed followed by addition of 15 ul of 20 mg/ml Proteinase K and incubated at 60 °C overnight. After digestion lysates were centrifuged, transferred to Phase lock separation tube and equal volume Phenol/chloroform/IAA was added. After centrifugation, aqueous phase was transferred to new tube and equal volume of Isopropyl alcohol was added. Tubes were inversed and centrifuges to precipitate DNA. The DNA pellet was washed with 75% alcohol and resuspended in 0.1× TE after air drying for 15 min. DNA concentration was measured and Taqman PCR based viral genome quantitation was performed. Primers (T-BGH-F: 5′-TCTAGTTGCCAGCCATCTGTTGT-3′; T-BGH-R: 5′-TGGGAGTGGCACCTTCCA-3′) and probe (T-BGH-PB: 5′-/56-FAM/TCCCCCGTGCCTTCCTTGACC/36-TAMNph/-3′) were diluted to100um and for final concentration of 500 nM and 200 nM respectively. DC67/+ SV40 DNA linearized with BglII diluted in TE buffer at various concentrations were used as standards.

#### Liver histopathology

Tissues were fixed in 10% neutral buffered formalin at the time of collection and processed as described in Singh et al.^29^ The 5um paraffin embedded sections were stained with hematoxylin and eosin (H&E) were evaluated semi quantitatively for inflammatory cells (plasma cells, lymphocytes and rare macrophages) by a board-certified pathologist.

### Serum chemistry analyses

Sera samples were analyzed for ALT and AST using the Randox Daytona Clinical Chemistry analyzer. The results were obtained for ALT (kit catalog AL3875) and AST (kit catalog AS3876) by the Tris buffer without P5P 37 °C method for both analytes. This is a UV method that is used for the quantitative determination of these analytes. The principle of the AST reaction is α-oxoglutarate reacting with l-aspartate in the presence of AST that forms l-glutamate and oxaloacetate. The indicator reaction utilized the oxaloacetate for a kinetic determination of NADH consumption. The principle of the ALT reaction is α-oxoglutarate reacting with l-alanine in the presence of ALT that forms l-glutamate and pyruvate. The indicator reaction utilized the pyruvate for a kinetic determination of NADH consumption.

### Nest building behavior assay

Nest building assay was adapted from Deacon et al.^44^ with minor modifications described by Singh et al.^29^.

### Liver RNA extraction, library preparation and sequencing

RNA from liver was isolated using the Qiagen RNeasy Lipid Tissue Mini Kit according to the manufacturer’s standard protocol for tissue with the added DNASE on-column digest step. RNA quantification and quality assessment were performed using a Nanodrop 8000 (Thermo-Fisher) and a Tapestation 4200 High Sensitivity RNA Screen Tape (Agilent), respectively. Total RNA was used as template prior to Oligo(dT) priming from Illumina’s TruSeq Stranded mRNA Library Prep Kit, according to manufacturer’s standard protocol. In short, mRNA was captured via oligoT labeled beads followed by conversion to first strand cDNA using SuperScriptII Reverse Transcriptase. Strand specificity was retained by incorporating dUTP instead of dTTP during the second strand cDNA synthesis (becomes quenched during amplification). The 3′ ends then are adenylated and Illumina Index adaptors were ligated onto the ends. A final PCR was performed to enrich fragments containing proper ligated ends. Integrity of each sample library was assessed using the Agilent Tapestation High Sensitivity D1000 kit run on a Tapestation4200. Libraries were sequenced on an Illumina HiSeq using a 2× 150 bp paired end-run scheme and sequenced at a depth of ~ 35 million paired reads per sample.

RNA-seq analysis was performed using QIAGEN Omicsoft studio version 10.2.4.7. Pathway and network analysis were performed using Ingenuity Pathway analysis IPA (QIAGEN Inc., https://www.qiagenbioinformatics.com/products/ingenuitypathway-analysis).

Gene expression of selected genes were validated by qPCR. The primer sequences used are listed in Supplemental Table S1.

### Liver proteomics analysis

Liver samples were cut into pieces on dry ice and weighed. Then placed into pre-chilled Eppendorf tubes. Ten 2-mm zirconia beads were added to each frozen tissue on dry ice. Transfer tubes to wet ice and 10× w/v of water supplemented with Halt™ protease and phosphatase inhibitor cocktails (Cat. # 78440, Pierce Biotechnology, Rockford, IL) were added. The samples were homogenized in TissueLyser for 3 min at 30 Hz and at 4 °C. The homogenates were transferred to fresh, pre-chilled tube and the total protein contents were measured with BCA assay (BCA™ Protein Assay kit, Pierce Biotechnology, Rockford, IL). About 40 µg of each sample was transferred to protein lo-bind Eppendorf tubes, and then reduced, alkylated and digested with Lys-C (for overnight) followed by trypsin (for 2.5 h). RapiGest SF surfactant (Cat. # 186001861, Waters corporation, Milford, MA) was used to enhance protease digestion. The digestion was quenched with formic acid. The samples were incubated for 45 min at 37 °C and centrifuged at 22,000×*g* for 30 min at room temperature. The supernatants were transferred and were dried and re-suspended in 80 µl of 3% acetonitrile in 0.1% formic acid.

Mouse liver digests were analyzed by nanoACQUITY UPLC^©^ (Waters Corporation, Milford, MA) coupled to Q Exactive™ High Field (HF)-X Hybrid Quadrupole-Orbitrap™ mass spectrometer (ThermoFisher Scientific). About 3 uL (~ 1.5 ug) digests from each sample were loaded onto a trap column (180 µm × 20 mm) from Waters Corporation with 5 µm 100 Å C18 medium and washed using a flow rate of 10 µl/min with 99% HPLC-grade water/1% acetonitrile (ACN)/0.01% formic acid (FA) for 3 min. Peptides were separated using a reversed phase nanoACQUITY UPLC 1.8 µm HSS T3 (100 µm × 100 mm) analytical column from Waters using a 65-min LC method at a flow rate of 500 nl/minute. Column temperature was maintained at 40 °C using column heater attached with UPLC^©^. MS data were acquired with data-dependent acquisition (DDA). using one full MS scan followed by twenty MS/MS scans. The full scan MS spectra were collected over 375–1600 m*/z* range with a maximum injection time of 30 ms, a resolution of 120,000 at 200 m*/z* and Automation Gain Control (AGC) target of 3e6. Fragmentation of precursor ions was performed by high-energy C-trap dissociation (HCD) with the normalized collision energy of 29 eV. MS/MS of the peptide ions were acquired at a resolution of 15,000 at 200 m*/z* and AGC target of 1e^5^ where the spectra were collected at centroid mode.

#### Peptide/protein identification and quantification

Progenesis QI-P (Waters, Milford, MA) software and Scaffold (ver. 4, Proteome Software, Inc., Portland, OR) was used to analyze and align the DDA raw data files. The false discovery rate (FDR) was set to 1% at peptide precursor as well as protein level. Data generated from Progenesis QI-P in .csv format was imported into OmicSoft Studio transferred (QIAGEN) to process further for statistical analysis and visualization.

### Statistical analysis

Statistical analysis was performed using the GraphPad prism software version 8.02. Blood Phe and Tyr (Fig. 1B,C, Supplementary Fig. S1A,B) were analyzed by Mixed- effects model followed by Tukey’s multiple comparison. One-way ANOVA nonparametric Kruskal–Wallis test and Dunn’s multiple comparison test was applied to the nesting assay (Fig. 4). The remaining data sets were analyzed using ordinary One-way ANOVA Tukey’s multiple comparison.

## Supplementary Information

Supplementary Information.
