# Supplementary material for: Blood phenylalanine reduction reverses gene expression changes observed in a mouse model of phenylketonuria
Source: Sci Rep. 2021 Nov 24;11:22886. doi: 10.1038/s41598-021-02267-2 (PMC8613214; doi:10.1038/s41598-021-02267-2)
Supplement: Supplementary file 1 — Supplementary Information. [file 41598_2021_2267_MOESM1_ESM.docx]

**Blood phenylalanine reduction reverses gene expression changes observed in a mouse model of phenylketonuria**

*Rachna Manek^1^, Yao V Zhang^1^, Patricia Berthelette^1^, Mahmud Hossain^2^, Cathleen S. Cornell^1^, Joseph Gans^2^, Gulbenk Anarat-Cappillino^3^, Sarah Geller^3^, Robert Jackson^1^, Dan Yu^1^, Kuldeep Singh^4^, Sue Ryan^4^, Dinesh S. Bangari^4^, Ethan Y. Xu^2,5^, Sirkka RM Kyostio-Moore^1^

^1^ Genomic Medicine Unit, Sanofi, Framingham, MA,^2^ Translational Sciences, Sanofi, Framingham, MA, ^3^ Pre-Development Sciences NA, Analytical R&D, Sanofi, Framingham, MA, ^4^ Global Discovery Pathology, Sanofi, Framingham, MA. ^5^ Excision BioTherapeutics, Cambridge, MA.

**
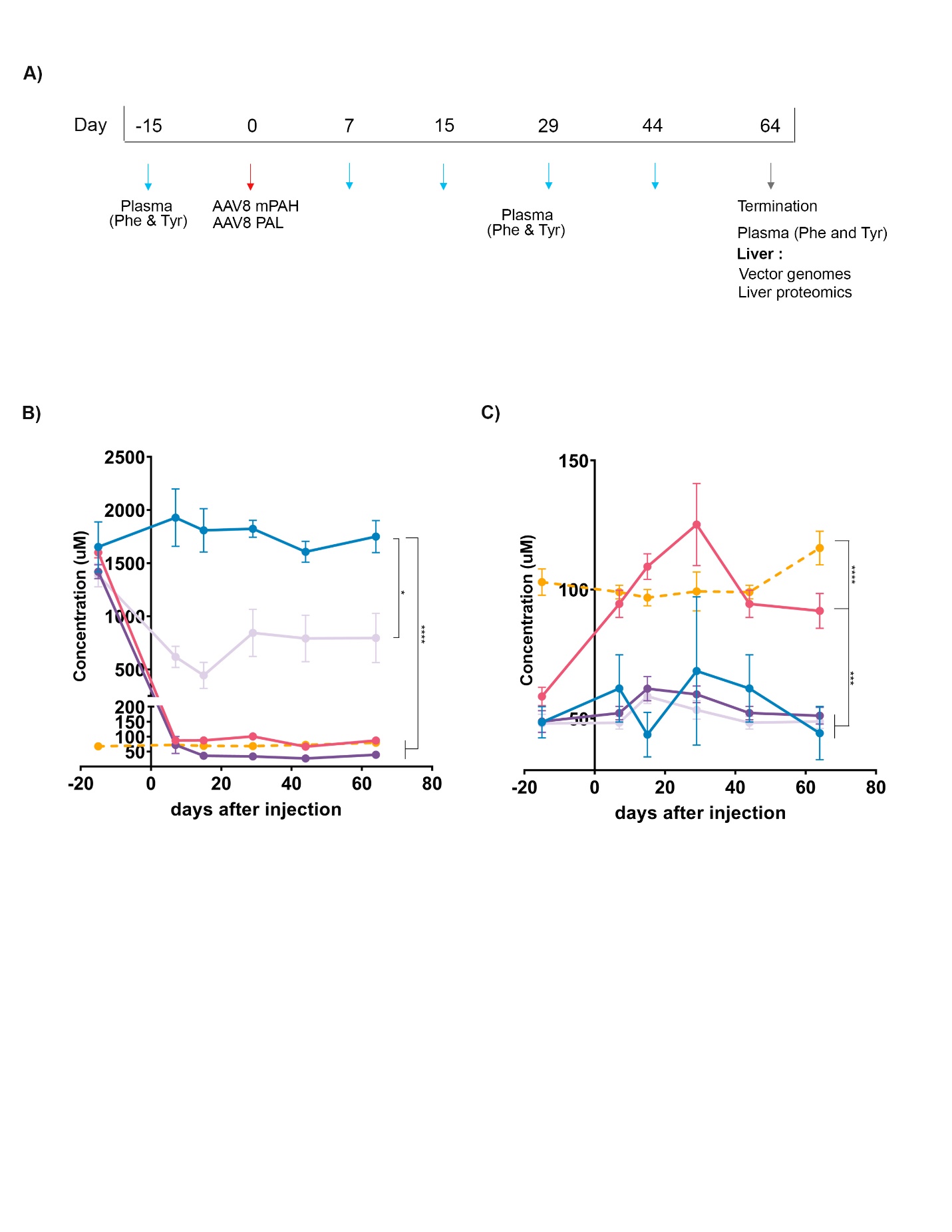
**

**Supplemental Figure 1.** **Titrating AAV PAL for treatment of PAH^enu2^ mice.**


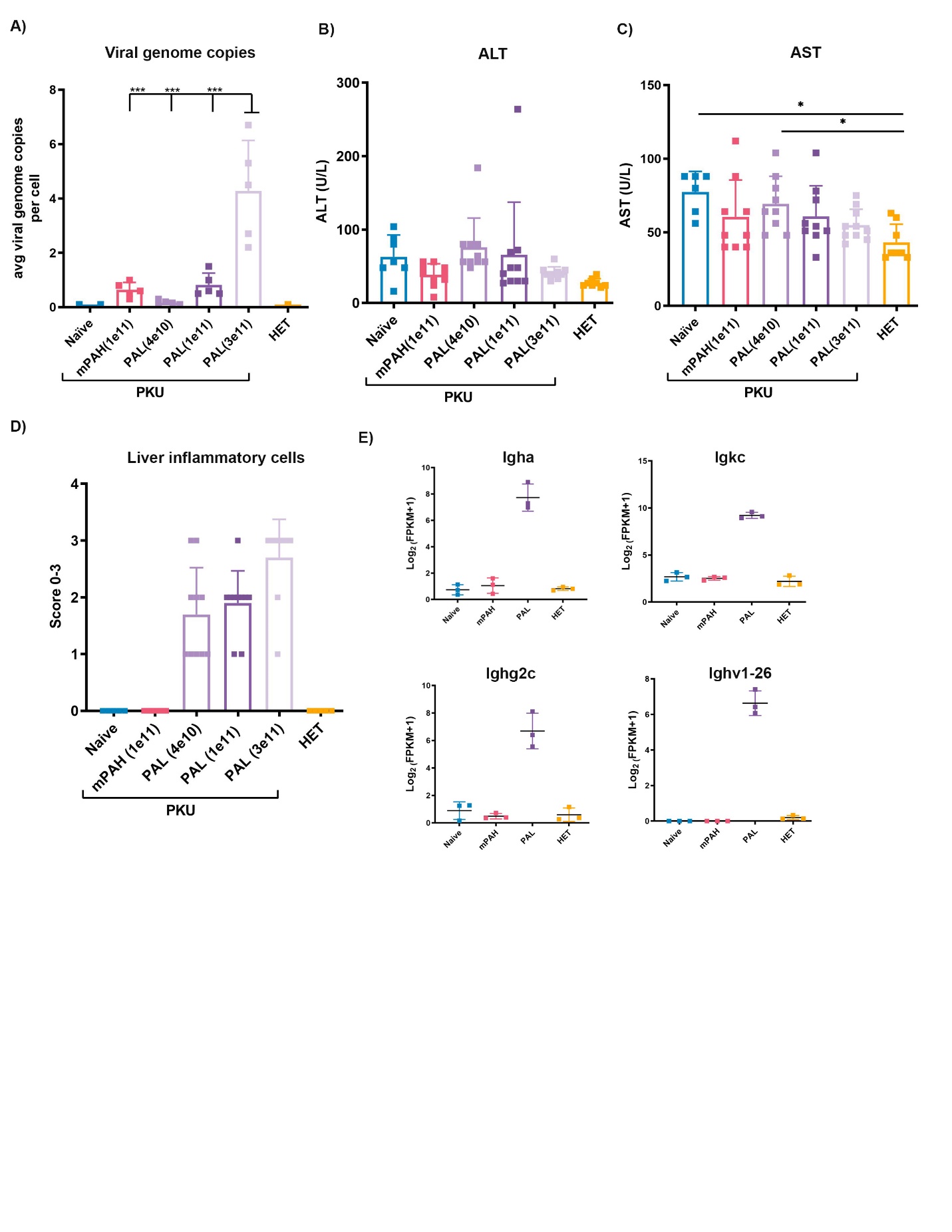


**Supplemental Figure 2.** **The effect of AAV PAL for treatment on livers of PAH^enu2^ mice.**

**
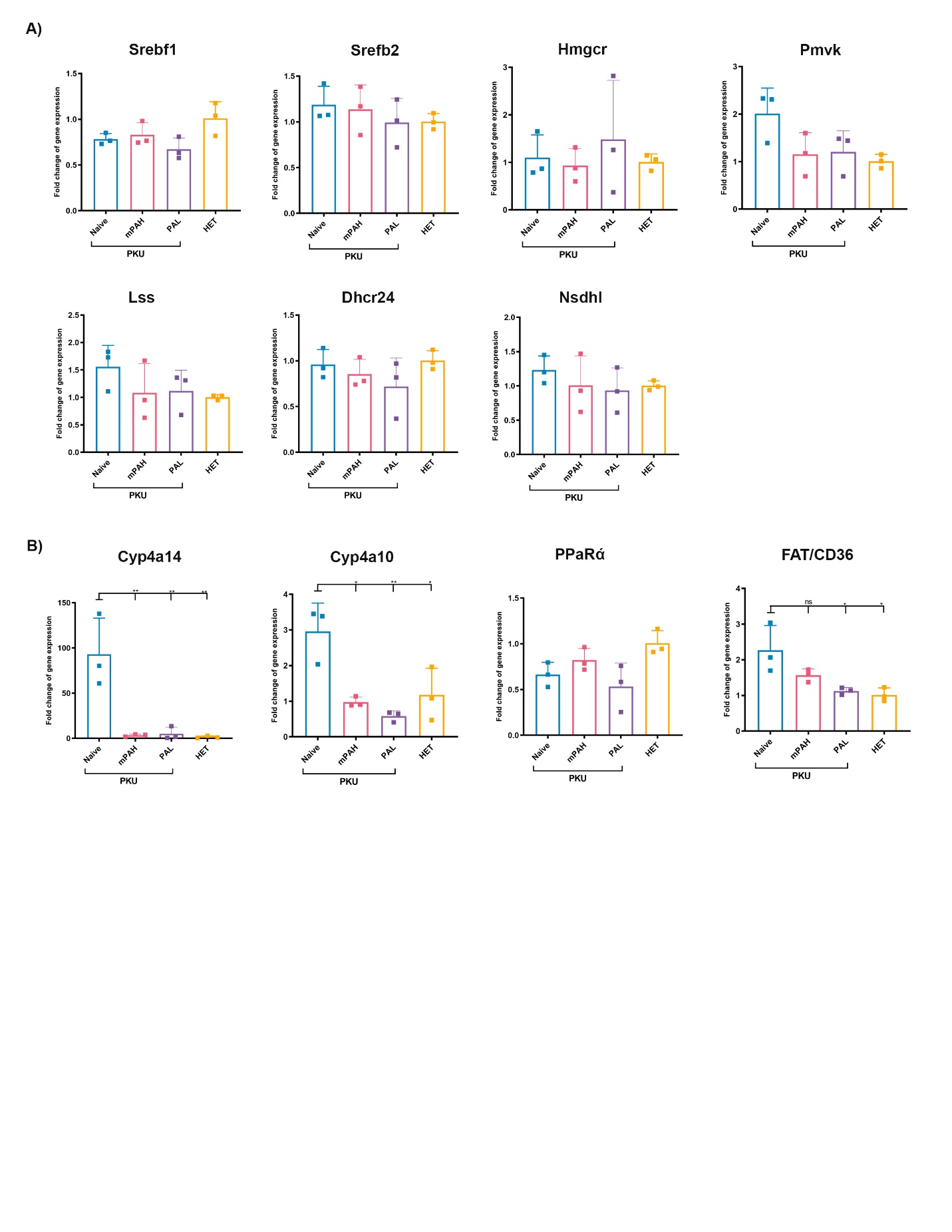
**

**Supplemental Figure 3. qRT-PCR validation of genes**


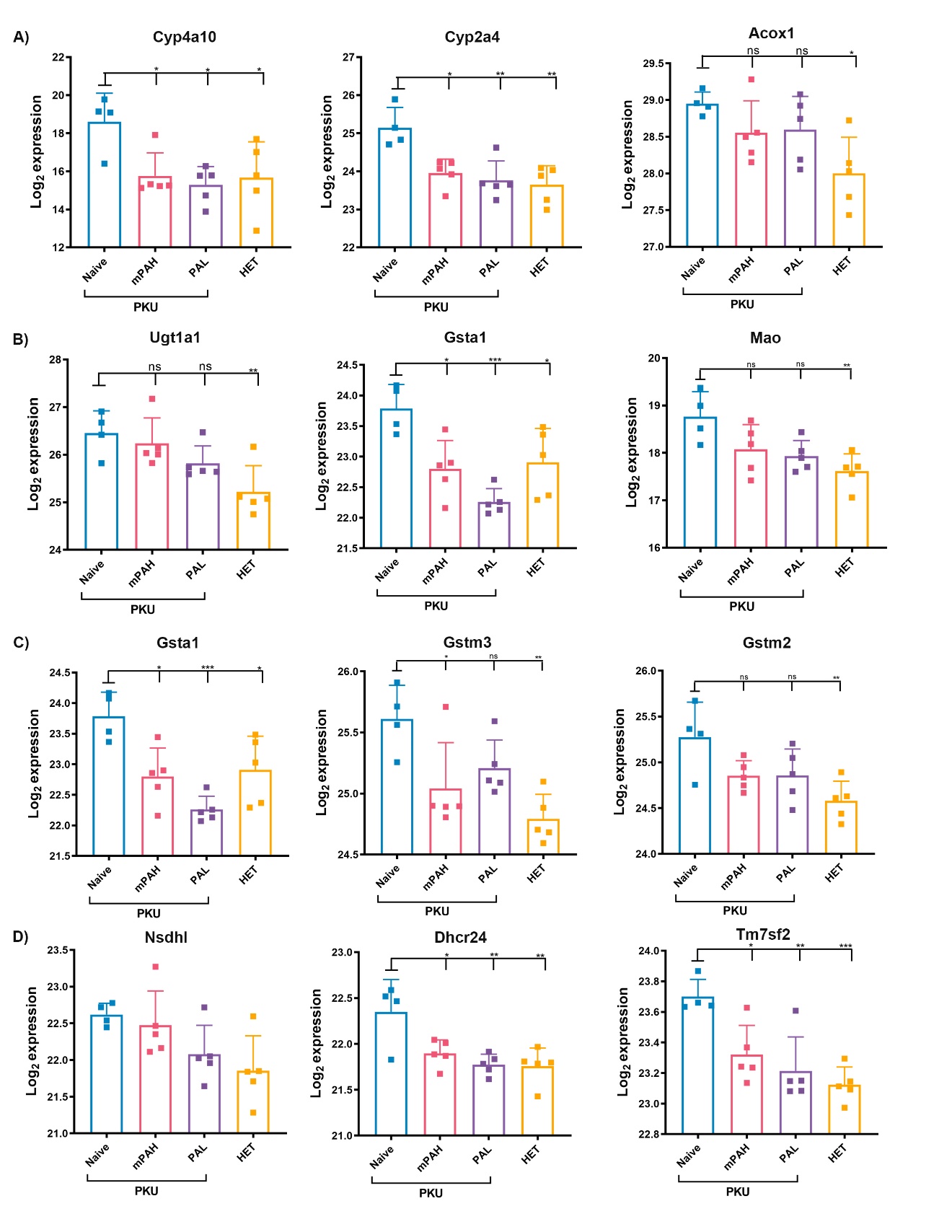


**Supplemental Figure 4 Proteins of top 3 affected pathway and cholesterol biosynthesis pathway.**

| Gene Symbol | Gene Name | Fisher Cat# | Dye |
| --- | --- | --- | --- |
| Pmvk | Phosphomevalonate Kinase | Mm01212763_m1 | FAM-MGB |
| Lss | Lanosterol synthase | Mm00461312_m1 | FAM-MGB |
| Nsdhl | Sterol-4-alpha-carboxylate 3-dehydrogenase | Mm00477897_m1 | FAM-MGB |
| Dhcr24 | Sterol delta(24) reductase | Mm00519071_m1 | FAM-MGB |
| CD36 | FAT (Fatty Acid Translocase) | Mm00432403_m1 | FAM-MGB |
| PPaRa | peroxisome proliferator activated receptor alpha | Mm00440939_m1 | FAM-MGB |
| Hmgcr | 3-hydroxy-3-methylglutaryl-Coenzyme A reductase | Mm01282499_m1 | FAM-MGB |
| Srebp1 | sterol regulatory element binding transcription factor 1 | Mm00550338_m1 | FAM-MGB |
| Srebp2 | sterol regulatory element binding transcription factor 2 | Mm01306292_m1 | FAM-MGB |
| Cyp4a10 | cytochrome P450, family 4, subfamily a, polypeptide 10 | Mm01622743_g1 | FAM-MGB |
| Cyp4a14 | cytochrome P450, family 4, subfamily a, polypeptide 14 | Mm00484135_m1 | FAM-MGB |
|  |  |  |  |
| Reference Gene | Gene Name | Cat # | Dye |
| Tbp* | TATA box binding protein | Mm01277042_m1 | Vic-MGB |

**Supplemental Table 1 List of Taqman assays used for qRT-PCR validation.**

**Supplemental Figure 1.** **Titrating PAL vector for treatment of PAH^enu2^ mice.** (**A)** Blood Phe levels and **(B)** blood Tyr levels were measured in naïve PAH^enu2^ mice. In a separate study, PAHenu^2^ mice were administered with PAH (1e11 vg) or PAL (1e10 vg and 1e11/mouse) to evaluate dose-response of PAL vector. A low dose of 1e10 vg of PAL vector did not normalize blood Phe levels while a dose of 1e11 vg overcorrected Phe levels. Analysis was performed with n=8-10/group. Group abbreviations: PKU naïve, untreated PAH^enu2^ mice, PKU PAH or PAL, treated PAH^enu2^ mice; HET, untreated HET mice. Two-way ANOVA Mixed effect analysis was performed, * p <0.05 and *** p < 0.001.

**Supplemental Figure 2.** **The effect of PAL expression in livers of treated PAH^enu2^ mice.** (**A)** Vector DNA levels in livers. Data demonstrated dose-response for PAL vector DNA as well as equal PAL and PAH vector DNA levels when comparable vector dose was delivered (1e11 vg/mouse). **(B)** Liver enzyme ALT showed no significant differences in treatment groups at day 42, **(C)** Liver enzyme AST showed no significant differences between treatment groups but showed significantly elevated AST levels in untreated PAH^enu2^ mice as compared to HET mice (HET). **(D)** Elevated levels of inflammatory cells were detected in all PAL treatment groups. Quantitation was performed on H&E-stained liver sections (scoring: 0 = none to rare inflammatory cells, 2 = rare small aggregates of inflammatory cells, 3 = few small aggregates of inflammatory cells, 4= multiple small aggregates of inflammatory cells) **(E)** RNA-seq analysis confirmed increased expression of various immunoglobin genes. Analysis was performed with n=8-10/group (RNAseq with n=3/group). Group abbreviations: PKU naïve, untreated PAH^enu2^ mice; PKU PAH or PAL, treated PAH^enu2^ mice; HET, untreated HET mice.

**Supplemental Figure 3. qRT-PCR validation**. Validation of genes involved in **(A)** cholesterol biosynthesis and **(B)** Cy4a proteins, upstream activator Peroxisome proliferator-activated receptor alpha (PPaRα) and downstream effector Fatty acid translocase (FAT)/ CD36. N=3 per group were used for analysis. Group abbreviations: PKU naïve, untreated PAH^enu2^ mice; PKU PAH or PAL, treated PAH^enu2^ mice; HET, untreated HET mice. One-way ANOVA Tukey’s multiple comparison, *p <0.05 and ** p<0.01.

**Supplemental Figure 4. Proteins of top 3 affected pathway and cholesterol biosynthesis pathway.** The top three proteins that change in **A)** LPS/IL-1mediated inhibition of RXR function, **B)** Xenobiotic metabolism PXR signaling pathway, **C)** Glutathione mediated detoxification and D) Cholesterol biosynthesis pathway. N=5 per group were used for analysis. Group abbreviations: PKU naïve, untreated PAH^enu2^ mice; PKU PAH or PAL, treated PAH^enu2^ mice; HET, untreated HET mice. One-way ANOVA Tukey’s multiple comparison *p <0.05 and ** p<0.01.
